# Sequence Determinants of G-Quadruplex Thermostability: Aligning Evidence from High-Precision Biophysics and High-Throughput Genomics

**DOI:** 10.3390/biom15111632

**Published:** 2025-11-20

**Authors:** Ke Xiao, Jiye Fu, Rongxin Zhang, Jing Tu

**Affiliations:** 1 State Key Laboratory of Digital Medical Engineering, School of Biological Science and Medical Engineering, Southeast University, Nanjing 211189, China; kexiao@seu.edu.cn (K.X.);; 2 Robert Lurie Comprehensive Cancer Center, Department of Obstetrics and Gynecology, Feinberg School of Medicine, Northwestern University, Chicago, IL 60611, USA

**Keywords:** G-quadruplex, thermostability, sequence features, biophysical assay, high-throughput sequencing

## Abstract

G-quadruplexes (G4s) are non-canonical nucleic acid structures that function as key regulatory elements in crucial cellular processes. Their biological functions are intrinsically linked to thermostability, which is governed by specific sequence features. This review systematically synthesizes evidence from high-precision biophysical studies and high-throughput genomic assays to delineate the sequence determinants of G4 thermostability. Analyses align the trends derived from both methodological paradigms and establish that stability emerges from a complex interplay among three structural elements: the G-tract core, whose length and integrity generally govern stability despite notable exceptions such as the anomalous stability of short G-tracts with 1-nt loops and the stabilization induced by large, structured bulges; the loops, which exhibit a consistent inverse relationship between length and stability across methods, though with context-dependent compositional effects and methodological disparities; and the flanking sequences, whose composition modulates stability and can bias topological outcomes. By integrating findings across scales, this work provides a unified conceptual framework connecting biophysical measurements with genomic observations—a critical step toward computationally predicting G4 stability, topology, and function directly from sequence, thereby advancing the understanding of their roles in health and disease.

## 1. Introduction: The Challenge of Quantifying G-Quadruplex Stability Across Scales

### 1.1. The Biological Significance of G4 Stability: From Biophysical Property to Functional Determinant

G-quadruplexes (G4s) are not merely structural curiosities but are widely recognized as functional regulatory elements whose biological roles are intrinsically linked to their stability [1,2,3,4]. These four-stranded nucleic acid structures, formed by the folding of guanine (G)-rich sequences, are broadly involved in a range of critical cellular processes, including the regulation of gene expression, DNA replication, telomere maintenance, and the preservation of genomic integrity [1,5,6,7]. Genomic analyses have revealed that potential G-quadruplex sequences (PQS) are not randomly distributed but are significantly enriched in key functional regions such as gene promoters, 5′ untranslated regions (5′ UTRs), telomeres, and replication origins [1,8,9,10]. In these contexts, G4 structures can function as physical barriers to enzymatic machinery like DNA or RNA polymerases or serve as recognition sites for specific regulatory proteins, thereby facilitating precise control over genetic activity [6,11].

The thermostability of a G4 structure—quantified by its resistance to thermal denaturation—directly governs its persistence and abundance within the cellular environment, which in turn determines the strength and duration of its biological function [3,12]. A highly stable G4 can act as a persistent “molecular switch” [12,13,14,15], whereas a less stable structure may form transiently to participate in dynamic regulatory events [15,16,17]. This correlation between stability and function has positioned G4s as attractive therapeutic targets, particularly in oncology [18,19,20]. For instance, stabilizing G4 structures in oncogene promoters or telomeric regions using small-molecule ligands can effectively suppress cancer cell proliferation and invasion [6,21].

The critical link between G4 stability and function is further underscored by evolutionary biology. A genome-wide study by Guiblet et al. revealed a pivotal pattern: G4 sequences located within functional genomic elements (e.g., promoters, UTRs, and replication origins) are not only overrepresented but also exhibit significantly higher thermostability and are under purifying selection. In contrast, unstable G4 motifs in regions like the non-transcribed strands of exons evolve neutrally, implying they are either non-functional or potentially deleterious [12]. Parallel evidence from the earlier work of Puig Lombardi et al. demonstrated negative selection against extremely stable G4 structures, which mitigates their adverse effects on genome integrity while preserving beneficial G4-related functions [22]. Together, these findings from both groups converge to suggest that G4 thermostability is not merely a random physicochemical trait, but a parameter tuned by evolution to fulfill specific biological roles. Consequently, the accurate quantification and prediction of G4 stability have progressed from a biophysical characterization to a central question in functional genomics.

### 1.2. The Methodological Divide in Stability Assessment

The current assessment of G4 stability relies on two distinct methodological paradigms, which differ fundamentally in their precision, throughput, and the nature of the information they yield, creating a significant methodological divide.

The first paradigm encompasses high-precision, low-throughput biophysical techniques. These methods focus on the precise characterization of individual, well-defined oligonucleotide sequences in vitro. Representative techniques include UV melting [23,24], circular dichroism (CD) [25], differential scanning calorimetry (DSC) [26], fluorescence resonance energy transfer (FRET) [27,28] and molecular dynamics (MD) simulations [7]. These techniques yield direct, quantitative thermodynamic parameters. The most common metric is the melting temperature (T_m_), at which 50% of the G4 structures denature into single strands. By analyzing the melting curve, one can also derive a suite of precise thermodynamic parameters, including the Gibbs free energy change (ΔG), enthalpy change (ΔH), and entropy change (ΔS). The principal advantage of these methods is the directness and precision of the measurements, establishing them as the “gold standard” for G4 stability assessment. However, their critical limitation is extremely low throughput, as each experiment typically analyzes only a single or a few sequences, precluding their application on a genome-wide scale.

The second paradigm consists of high-throughput, indirect genomic methods. These techniques leverage the obstructive effect of G4 structures on DNA polymerase, coupled with high-throughput sequencing. The most prominent example is G4-Seq [8,29]. In a standard G4-Seq experiment, DNA polymerase progression is challenged under G4-stabilizing conditions (e.g., in the presence of K^+^ or specific ligands). A stable G4 on the template strand acts as a physical barrier, inducing polymerase pausing or dissociation [8,11]. This stalling event manifests in sequencing data as a sharp decline in read quality and a significant increase in the base mismatch rate (Mismatch Percentage, MM%) immediately downstream (3′) of the G4 initiation site [8]. Furthermore, Tu et al. (2021) developed the G4-miner algorithm, which infers G4 formation by detecting subtle fluctuations in sequencing quality scores from standard whole-genome resequencing data [30]. Thus, stability is inferred indirectly: a higher MM% or a more pronounced quality drop is interpreted as evidence of a more stable G4 that more effectively impedes the polymerase [8,30]. The unparalleled advantage of these methods is their throughput, enabling the mapping of hundreds of thousands of potential G4 structures across an entire genome in a single experiment. Their limitation, however, is that the sequencing signal is an indirect proxy for stability, not a direct thermodynamic measurement, and can be confounded by other factors.

### 1.3. Focusing on Sequence Determinants

An “alignment problem” exists between these two methodological classes. They measure fundamentally different physical quantities: biophysical methods like UV melting assess a thermodynamic equilibrium property (T_m_), whereas high-throughput sequencing assays measure the kinetics of an enzymatic process (polymerase stalling efficiency). While a thermodynamically more stable G4 is likely to present a stronger kinetic barrier, this relationship is not guaranteed. Factors such as G4 unfolding kinetics, specific interactions with the polymerase, and local structural topology can independently influence pausing efficiency, decoupling it from the overall T_m_.

Researchers have provided initial clues to help bridge this divide. For instance, Wang et al. demonstrated that enzyme-G4 interactions, such as Pif1-mediated unwinding, are sensitive to G4 stability and are thus influenced by structural features like loop length [31]. Similarly, Sahakyan et al. confirmed that the MM% values from G4-Seq correlate with the biophysically assessed stability (via CD, ^1^H NMR, and UV melting) of selected G4 sequences [32]. However, systematically validating the relationship between thermodynamic stability and high-throughput sequencing signals (e.g., MM% from G4-Seq or quality scores from G4-miner) across an entire genome, with its millions of G4 motifs, remains a formidable challenge.

A pragmatic approach to this problem is to compare the readouts from both experimental paradigms, observing whether they exhibit consistent relative changes in response to identical sequence features. If consistent correlative patterns emerge, it would suggest that both methods capture a core, shared attribute of G4 stability. Establishing such a correspondence would greatly facilitate the extrapolation of G4 stability understanding and its application across the entire genome. Therefore, this review aims to collate and integrate evidence from both fields to provide a theoretical foundation for connecting them.

Before delving into sequence features, it is crucial to acknowledge that G4 stability is profoundly influenced by the intracellular environment. For instance, K^+^ generally stabilizes G4s more effectively than Na^+^. This preference is driven by the greater energetic cost of dehydrating Na^+^; although Na^+^ binding to a quadruplex is thermodynamically more favorable, the dehydration penalty for Na^+^ far exceeds that for K^+^ [4]. Furthermore, the crowded intracellular environment (macromolecular crowding) preferentially stabilizes the compact, folded state of G4s through the excluded volume effect, thereby promoting their formation and stability [33,34,35]. Detailed discussions on these topics can be found in reviews by Jana and Weisz [36] and Nishio et al. [37].

**Figure 1:**
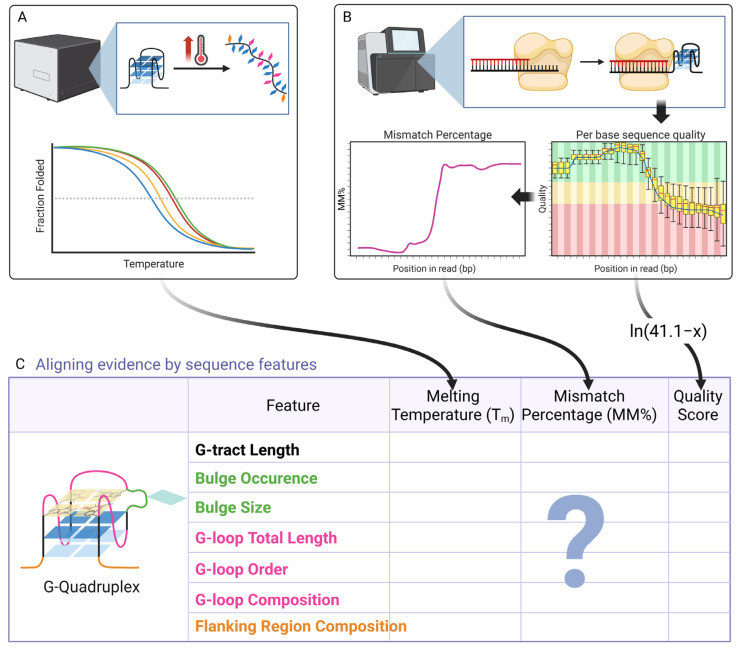
Conceptual framework for aligning evidence from high-precision biophysical and high-throughput sequencing methods. (**A**) High-precision biophysical methods, exemplified by UV melting and CD melting assays, primarily yield thermodynamic parameters such as melting temperature (T_m_). (**B**) High-throughput sequencing methods, such as G4-Seq and G4-miner, provide genome-wide metrics including base mismatch percentage (MM%) and sequencing quality scores. The original quality scores were transformed to ensure that higher values correspond to greater G4 stability, consistent with the interpretation of MM%. (**C**) Readouts from both methodological paradigms were systematically compared and aligned to determine whether they exhibit consistent responses to identical sequence features, as illustrated by six representative sequence features analyzed in this study.

While these environmental factors provide the essential context for in vivo G4 formation, this review will deliberately focus on elucidating the intrinsic sequence features that determine G4 thermostability under constant, well-defined experimental conditions (typically in K^+^-containing buffer). By systematically isolating and analyzing the influence of core sequence elements—the G-tracts, loops, and flanking regions—this study aims to synthesize the empirical rules that link sequence features to stability data, establish a set of generalizable principles by examining both consistencies and discrepancies between the two approaches (Figure 1), and bridge the findings from low-throughput biophysical methods and high-throughput genomic assays.

For each sequence feature, studies based on high-precision biophysical methods were reviewed, typically focusing on T_m_ trends derived from assays such as UV melting and CD melting (Figure 1A). Accompanying explanations for these trends—including topological alterations, conformational details, and associated energy changes—were also compiled. These T_m_-based trends were subsequently compared and aligned with corresponding data from high-throughput sequencing methods, specifically Mismatch Percentage (MM%) from G4-Seq and sequencing quality scores from G4-miner (Figure 1B, Appendix A). To ensure consistent interpretation across metrics, the original G4-miner quality scores (x) were transformed using the function ln(41.1 − x). This transformation was reasonable as the native Phred + 33 quality scores (range: 0–41) are inversely correlated with sequencing error rates. The operation 41.1 − x reverses this relationship to generate positive values, while the subsequent natural logarithm enhances discrimination near the maximum score, i.e., 41, thereby aligning the metric with MM% where higher values indicate greater G4 stability.

## 2. The G-Tract Core: The Impact of Size and Imperfections on Stability

The G-tracts that constitute the core of the G-quadruplex are fundamental to its structural integrity [38,39]. This section examines how the length of these G-tracts and their sequence integrity—specifically, the presence of non-guanine base insertions that create bulges—influence the overall stability of the G4 structure.

### 2.1. G-Tract Length: Incomplete Monotonic Relationship with Stability

Intuitively, longer G-tracts would be expected to confer greater stability due to the stacking of additional G-tetrad layers. However, experimental evidence reveals that the relationship between G-tract length and G4 stability is not a simple monotonic progression. Instead, it is strongly modulated by the length of the connecting loops, exhibiting complex, incomplete monotonic characteristics [40].

The research by Rachwal et al. provides a demonstration of this phenomenon. They compared the stability of two sequence series: one with G-tracts connected by a single thymine (T). For these d(G_n_T)_4_ sequences, the stability order was unexpectedly nonmonotonic: n=3 > n=7 > n=6 > n=5 > n=4. The sequence with the shortest G-tracts, d(G_3_T)_4_, exhibited “anomalous stability,” possessing a T_m_ significantly higher than counterparts with longer G-tracts (G_4_, G_5_, G_6_). In contrast, when the loop length was increased to two thymines (T2) in the case of d(G_n_T_2_)_4_, the stability trend returns to the intuitive pattern, increasing monotonically with G-tract length: n=7 > n=6 > n=5 > n=4 > n=3.

The “anomalous stability” of d(G_3_T)_4_ underscores a delicate balance between two opposing forces: the enthalpic gain from increased G-tetrad stacking and the entropic/steric cost imposed on the connecting loops. While each additional G-tetrad layer contributes favorable enthalpy, in an intramolecular G4 connected by the very short (1-nt) V-shaped loops, longer G-tracts force these loops to span a greater spatial distance. This imposes significant conformational strain on the phosphodiester backbone, offsetting the energetic benefits of extra tetrads. This interpretation will be supported by later discussions on loop length (Section 3.1), wherein short loops favor parallel topologies due to steric constraints, while longer loops afford the flexibility for alternative antiparallel or hybrid folds [41,42,43].

### 2.2. Bulges in G-Tracts: The Impact of Imperfections

Although the canonical G4 model requires continuous G-tracts, accumulating evidence demonstrates that G4s with structural imperfections in their core are not only feasible but also biologically significant. These deviations include bulges (non-guanine nucleotides inserted within a G-tract) [44,45], vacancies (missing guanines in a G-tetrad) [46], and dynamic fluctuations like strand register shifts [47]. Rather than being mere structural defects, these features expand the structural diversity and functional repertoire of G4s.

Early foundational work [48] established that bulges generally reduce G4 stability, and they systematically investigated the effects of residue type, size, position, and number on G-quadruplex structure and stability. Their UV-melting experiments demonstrated that the number and size of bulges substantially diminish stability. This finding was later corroborated by Sarkar et al., who further attributed the destabilization of bulged G4s (buG4s) to an unfavorable enthalpic contribution during formation. They proposed that this arises from weakened base stacking interactions and compromised hydrogen bonding within the G-quartets due to the incorporated bulge [45].

The chemical identity of the bulge residue significantly influences its impact on stability. Mukundan and Phan observed a pronounced decrease in T_m_ values when the bulge was an adenine (A), suggesting that the lower stability might result from an increased solvation entropy associated with the exposure of its larger aromatic surface area [48]. Sarkar et al. confirmed this trend, reporting that the destabilization follows the order: A > C ≥ T. They suggested that this discrepancy is likely due to the larger steric hindrance imposed by an adenine bulge, which more severely perturbs G4 folding [45].

The influence of bulge position appears to be context-dependent. In the UV-melting assays conducted by Mukundan and Phan, the positional effect of a single bulge was minimal, with T_m_ variations of only about 1 °C across different locations [48]. In contrast, Sarkar et al. found that the destabilizing effect is indeed modulated by position; for instance, bulges at position 1 in the first G-tract were less destabilizing than at other positions. They suggested that bulges at other sites may increase the inter-quartet distance, thereby weakening stacking interactions and leading to position-dependent stability variations [45]. Furthermore, Zhang et al. indicated that bulge position not only influences G4 topology but also the kinetics and pathway of folding. In guanine-vacancy-bearing G-quadruplexes, which are key folding intermediates, the location of the vacancy can strongly dictate the stability of the structure [16].

Despite their inherent destabilizing effect, several compensatory structural mechanisms can mitigate, or in some cases reverse, the negative impact of bulges. For example, a bulge can form a structured hairpin stem that stacks onto an outer G-tetrad. In such Quadruplex–Duplex hybrids, a larger, structured duplex bulge can confer greater thermostability than an unstructured loop of the same sequence [49]. Environmental factors can also play a compensatory role. Sarkar et al. showed that under molecular crowding conditions, the stability of both canonical and bulged G4s increases, thereby reducing the relative destabilization caused by the bulge [45]. This suggests that the cellular environments may effectively “rescue” certain bulged structures that would be marginally stable under standard dilute in vitro conditions.

### 2.3. Aligning the Evidence: G-Tract and Bulge Features in High-Throughput Sequencing Analysis

**Figure 2:**
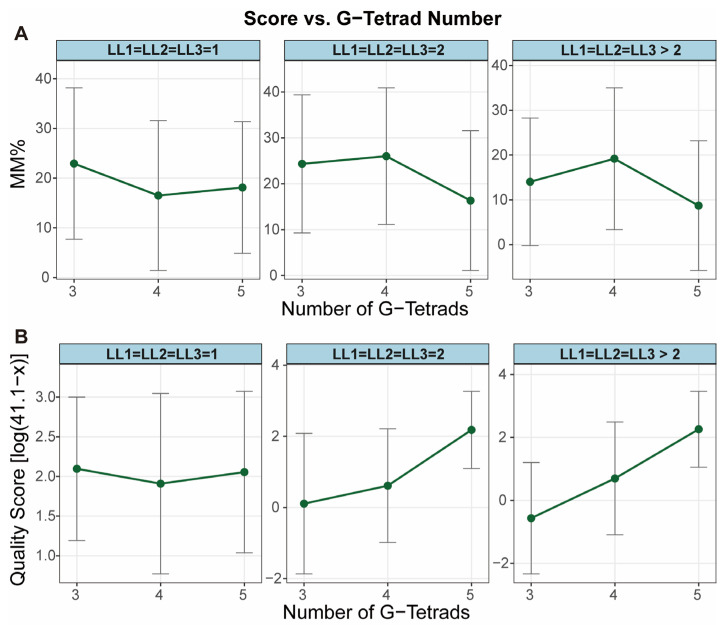
Dependence of G4 stability scores on the number of G-tetrad layers. Mean stability scores are plotted against the number of G-tetrad layers, with data stratified by loop length (1 nt, 2 nt, and >3 nt). (**A**) Mean MM% from G4-Seq. (**B**) Mean transformed quality score from G4-miner. The original G4-miner quality score (x) was transformed as ln(41.1 − x). Error bars represent standard deviations across samples. The terms LL1, LL2, LL3 represent lengths of loop 1, loop 2 and loop 3, respectively.

The influence of G-tract length on stability, as established by Rachwal et al. [40], is largely recapitulated by high-throughput sequencing data, both MM% from G4-Seq and quality score from G4-miner. In general, the inferred stability score increases with G-tract length, with a notable exception for three-layer G4s connected exclusively by 1-nt loops (Figure 2). The G4-miner algorithm successfully captured all documented trends, whereas G4-Seq accurately reflected the behavior of sequences with 1-nt loops but showed discrepancies in the case of 5-layer G4s when the loops exceeded 1 nt in length.

A possible explanation for the anomalous behavior of 5-layer G4s in G4-Seq may lie in the experimental design, which compares sequencing results under K^+^ versus Na^+^ stabilization conditions. Since 5-layer G4s exhibit inherently high stability in both ionic environments, the additional stabilizing effect conferred by K^+^ is relatively minor. Consequently, the MM% values—reflecting the differential stability between ionic conditions—are smaller for these structures compared to their counterparts with fewer G-tetrad layers.

**Figure 3:**
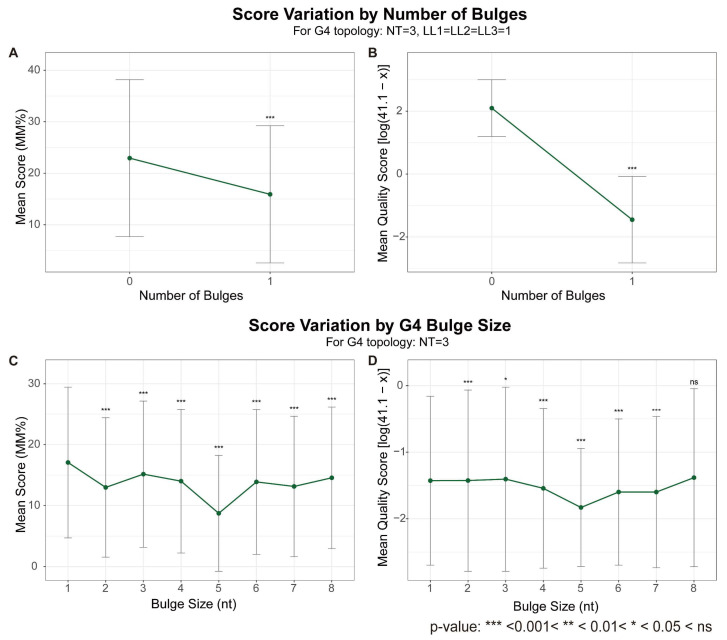
Influence of bulge presence and size on G4 stability scores. (**A**,**B**) Effect of bulge occurrence. Mean MM% from G4-Seq (**A**) and mean transformed quality score from G4-miner (**B**) are shown for three-layer G4s—with or without bulges—where all loops are 1 nt in length. (**C**,**D**) Effect of bulge size. Mean MM% (**C**) and transformed quality score (**D**) are shown for three-layer G4s with only 1-nt loops, grouped by bulge size (1 to 8 nt). The original G4-miner quality score (x) was transformed as ln(41.1 − x) in panels B and D. Error bars represent standard deviations. Significance was assessed using the Wilcoxon rank-sum test: in (**A**,**B**), between the two groups; in (**C**,**D**), between each bulge size and the first group (indicated by symbols above points). The terms LL1, LL2, LL3 represent lengths of loop 1, loop 2 and loop 3, respectively, and NT represents number of G-tetrads.

Both high-throughput methods are also capable of detecting the destabilizing influence of bulges on G4 stability. When compared to G4s with a bulge, G4s without bulges consistently yield higher stability scores in both G4-Seq and G4-miner analyses (Figure 3A,B). Furthermore, the impact of bulge size was correctly captured by both G4-Seq and G4-miner: stability levels decrease as the bulge size increases up to 5 nucleotides, confirming that bulges act as a general destabilizing factor within this size range. Intriguingly, for bulges larger than 5 nt, a positive correlation between stability metric and bulge size emerges. This reversal in trend likely reflects stabilization conferred by structured motifs, such as the formation of an internal duplex within the elongated bulge, which can compensate for the initial destabilization.

## 3. The Loops: The Impact of Length, Order and Nucleotide Composition on Stability

The loop regions connecting the G-tracts are critical structural components beyond the core G-tetrad. Their length, nucleotide composition, and sequential arrangement collectively form a fundamental “structural code” that dictates both the topology and thermostability of a G-quadruplex.

### 3.1. Loop Length: A General Inverse Correlation with Stability

Extensive biophysical evidence supports a fundamental principle: G4 thermostability exhibits a strong inverse correlation with the length of its connecting loops, considering both the total loop length and the length of any specific individual loop. As early as 2004, Phan et al. attributed the differential stability of two G4 structures from the *MYC* promoter to the size of their central loops, finding the variant with a two-residue loop to be more stable than the one with a six-residue loop [50]. Subsequently, Risitano and Fox demonstrated that the structure and stability of intramolecular quadruplexes are profoundly influenced by loop length and composition, using substitutions with non-nucleosidic linkers [51]. Moreover, an example by Fernando et al. about the c-kit21 G4 showed that the addition of a single nucleotide in the third loop can reduce the T_m_ by a remarkable 10 °C [52].

This principle was quantitatively defined by Guédin et al., who established an empirical rule by a systematic study on G4 loop length: each additional nucleotide in the total loop length reduces the T_m_ by approximately 2 °C in a K^+^ environment, corresponding to a loss in free energy (ΔG) of about 0.3 kcal/mol [53]. A broadly similar trend is observed in a Na^+^ environment, although the linear relationship between T_m_ and loop length is less pronounced.

From a thermodynamic perspective, the destabilizing effect of long loops is primarily entropy-driven. A longer, more flexible loop possesses greater conformational entropy in the unfolded, single-stranded state. Upon folding into a G4 structure, this flexibility is constrained, leading to a significant entropic penalty. Hao et al. demonstrated that the longer the central loop, the greater this entropic cost, thereby exerting a more substantial destabilizing effect on the folded structure [54]. Although this general trend is robust, its impact is context-dependent. For example, a G4 structure containing two very short loops (e.g., 1-nt or 2-nt) can often tolerate a single, considerably longer third loop without a catastrophic loss of stability [53]. This observation suggests a synergistic effect among loops, whereby the high stability conferred by two short loops can partially compensate for the large entropic penalty imposed by one long loop.

Furthermore, loop length is a key determinant of G4 topology. Short loops, due to steric constraints, typically favor a parallel G-tract alignment [55]. In contrast, longer loops provide the flexibility necessary for the adoption of more complex antiparallel or hybrid topologies [33,41,42,43]. Additionally, the loop length may influence the formation of multimeric G4s [55]. A more detailed review about the influence of loops on G4 topology was provided by ref [36].

It is also noteworthy that under specific conditions, long loops can form stable secondary structures, such as hairpins. These can lead to the formation of Quadruplex–Duplex hybrids, which may mitigate, or in some cases even reverse, the intrinsic destabilizing effect typically associated with long loops [56,57,58,59,60].

### 3.2. Loop Permutation: The Critical Role of Sequential Order

The complexity of G4 sequence design is further exemplified by the phenomenon of loop permutation. Even when the G-tracts, total loop length, and nucleotide composition are held constant, merely altering the sequential order of the three loops can profoundly impact both the topology and thermostability of the resulting G4 structure [61,62,63].

The magnitude of this effect is substantial. A systematic investigation by Cheng et al. demonstrated that different loop permutations can alter the T_m_ by up to 17 °C—an effect comparable to that of modifying loop length or key base compositions by several nucleotides [62]. Agrawal et al. observed similar trends in their study of G4s within the *VEGF* promoter [64]. A central principle emerging from these studies, as proposed by Cheng et al., is the dominant role of the central (second) loop length in determining the final architecture. Sequences with a longer central loop exhibit a strong propensity to adopt stable non-parallel (hybrid or antiparallel) topologies, whereas those with a shorter central loop typically form parallel topologies, which are often less thermostable [62]. This finding is corroborated by Beniaminov et al., who showed that replacing TTA loops with 1-nt loops can shift the conformation of human telomeric G4s from a 3 + 1 hybrid type to a parallel form [65].

The significant influence of loop permutation underscores that loops are not merely passive linkers but play an active, guiding role in the G4 folding pathway, effectively functioning as a “topological code.” This effect cannot be fully explained by the intrinsic thermodynamic properties of the loops alone; it must also involve the geometric constraints and kinetic choices encountered by the single strand during its folding process. Supporting this view, Chen et al. reported a sequence with a 4T central loop that forms a structure exhibiting cation-sensitive G-switch behavior, highlighting how loop arrangement can encode a regulatory potential within the folding landscape [63].

### 3.3. Loop Nucleotide Composition: Contradictions Depending on Context

Beyond loop length, the specific nucleotide composition of the loops adds a further layer of complexity to G4 stability. Based on a study of quadruplex-forming sequences from the promoter regions of various proto-oncogenes, Kumar and Maiti observed that these naturally occurring quadruplexes exhibit a wide range of thermodynamic stabilities (ΔG from 1.7 to 15.6 kcal/mol) and suggested that both loop length and composition collectively influence structure and thermodynamics, making it challenging to establish generalized correlations based on length alone [66].

Nevertheless, certain general principles can be discerned. The identity of the bases within the loops fine-tunes stability through specific interactions, including base stacking, hydrogen bonding, and steric effects [67,68,69]. Research by Guédin et al. indicated that the presence of adenine is equally unfavorable in either the first or the last loop [67]. A destabilizing effect of adenine was also observed by Nagatoishi et al. in a two-layer G4 system [70]. Furthermore, a subsequent study by Guédin et al. demonstrated that in a three-base loop, an adenine at the first position immediately following a G-tract exerts a significantly more destabilizing effect compared to other bases [71]. This effect has been corroborated in the thermodynamic study of adenine-to-thymine substitutions in the G4 loops [72].

However, a notable counterexample is found in the human telomeric repeat sequence (TTAGGG)_n_. The TTA loops are evolutionarily conserved, and research by Tucker et al. has shown that in a Na^+^ environment, the adenines within these loops are critical for maintaining G4 stability. Systematic substitution of these adenines with thymines resulted in fluctuations in both melting temperature and unfolding enthalpy, indicating that adenine stabilizes the structure via stacking interactions with the adjacent G-tetrad and through the formation of intra-loop hydrogen bonds [73]. Further complicating the picture, Agrawal et al.’s study on *VEGF* promoter G4s revealed that G-to-T mutations of two residues in the central loops did not significantly alter T_m_ [64].

One possible explanation for these apparent contradictions could be: the contribution of any single base to stability might be dependent on its specific three-dimensional structural context. In the study of Tippana et al., the larger purine ring of adenine may introduce steric hindrance within the loop regions of parallel conformations, thereby disrupting optimal structural packing [43]. In the MD simulations by Zhu et al., the adenine in the TTA loop is precisely positioned to engage in favorable π-π stacking interactions with the terminal G-tetrad, thereby providing additional enthalpic stabilization [74]. Suggested by Lenarčič Živković et al., specific interactions mediated by loop adenines can drive significant structural changes [75], and thus influence the topology of G4s and act as a structural switch that narrows a broad range of putative folds into an antiparallel conformation.

This comparison underscores that any simple rule regarding base composition must be considered in conjunction with the likely topological context. It also implies that predicting the effect of loop composition may first require predicting the most probable topology—a feature that is itself influenced by loop length and arrangement. This interdependence creates a complex, multi-parameter optimization problem in G4 structure prediction [76].

### 3.4. Aligning the Evidence: Interpreting Loop Features in High-Throughput Data

**Figure 4:**
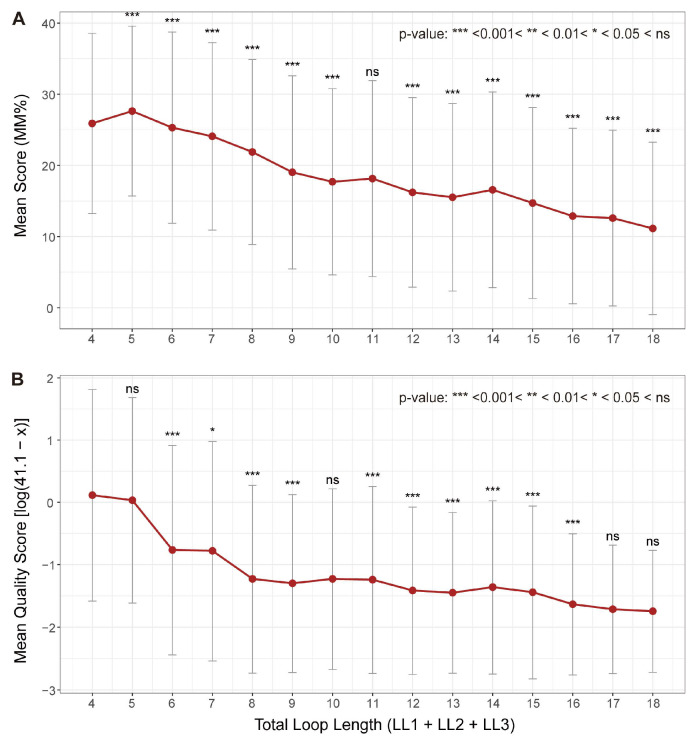
Variation in G4 stability scores with total loop length. Mean stability scores derived from (**A**) MM% in G4-Seq and (**B**) transformed quality scores from G4-miner are shown for G4s grouped by total loop length. Each point represents the mean of 1000 resampled sequences. To ensure that higher values correspond to greater stability—consistent with the interpretation of MM%—the original G4-miner quality score (x) was transformed as ln(41.1 − x). Error bars indicate standard deviations across loop length groups. Significance between adjacent loop length groups was assessed using the Wilcoxon rank-sum test; symbols above data points denote the statistical significance relative to the group immediately to the left. The terms LL1, lLL2, LL3 represent lengths of loop 1, loop 2 and loop 3, respectively.

The negative correlation between thermostability of a G4 structure and the total loop length is also discernible in both MM% from G4-Seq and quality score from G4-miner (Figure 4). Despite inherent data variability, the trends are clear: both methods show a decrease in indicated stability with increasing total loop length, consistent with the principles established by low-throughput biophysical techniques [50,51,52,53].

**Figure 5:**
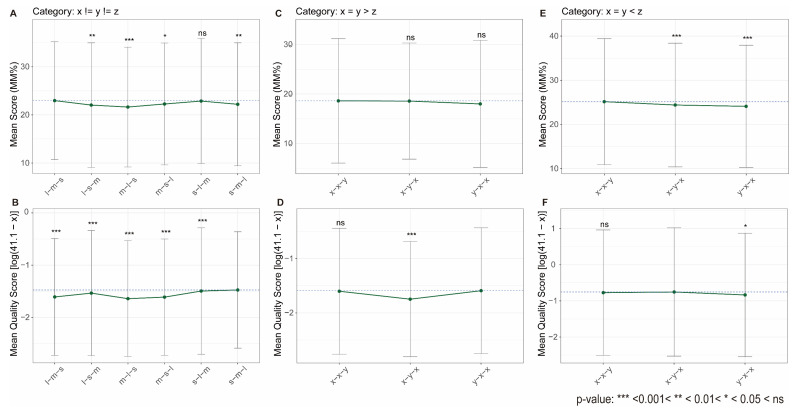
G4 stability score variation with loop permutation. Stability scores are shown for G4s grouped by different loop length permutations: (**A**,**B**) three unequal loop lengths (x≠y≠z), (**C**,**D**) two long loops of equal length and one shorter loop (x=y>z), and (**E**,**F**) two short loops of equal length and one longer loop (x=y<z). The upper panels (**A**,**C**,**E**) show the mean MM% from G4-Seq, while the lower panels (**B**,**D**,**F**) show the mean transformed quality score from G4-miner. The original G4-miner quality score (x) was transformed as ln(41.1 − x). Error bars represent standard deviations. Statistical significance was assessed using the Wilcoxon rank-sum test, comparing each group with the highest mean stability score within each panel.

We further compared the influence of loop permutation on stability scores from high-throughput methods with the findings of Cheng et al. [62]. For G4s with three unequal loop lengths (denoted as x≠y≠z), similar trends were partially observed (Figure 5A,B). For instance, the s-l-m (short-long-median) and l-s-m (long-short-median) permutations, corresponding to groups A2 and A5 in Cheng et al.’s study, generally exhibited high stability scores. Conversely, the m-l-s (median-long-short) permutation (group A4 in Cheng et al.) tended to yield lower scores, indicating destabilization of the G4 structures. However, discrepancies were noted, such as unexpectedly high scores for the l-m-s group in G4-Seq and the s-m-l group in G4-miner. For G4s with loop length combinations x=y>z and x=y<z, the scores from G4-Seq were inconsistent with Cheng et al.’s experimental results [62] (Figure 5C–F). In contrast, G4-miner scores better captured the expected trends; for example, the x-y-x permutation (group A2 in Cheng et al.) correctly showed the lowest or highest stability levels for the x=y>z and x=y<z combinations, respectively.

It should be noted that the original biophysical observations are highly context-dependent, as trends in T_m_ across loop permutations are not universally consistent (Table 1 in [62]), which may partly explain the variability in high-throughput data. Moreover, the magnitude of stability score differences attributable to loop permutation in sequencing-based methods is considerably attenuated compared to the ~17 °C T_m_ variations reported in biophysical studies, indicating that genomic assays are generally less sensitive to such structural nuances. This attenuated sensitivity aligns with earlier analyses based on high-throughput data and molecular dynamics simulations [77], which concluded that while total loop length exerts a clear negative effect on G4 stability, the specific distribution of loop lengths has minimal influence. This inherent limitation of sequencing-based approaches may explain not only the discrepancies with biophysical measurements but also the divergent outputs between G4-Seq and G4-miner, as the topological and stabilizing effects of K^+^ [36] employed in G4-Seq likely dominate the sequencing signal, thereby overshadowing the weaker modulation induced by loop permutation.

**Figure 6:**
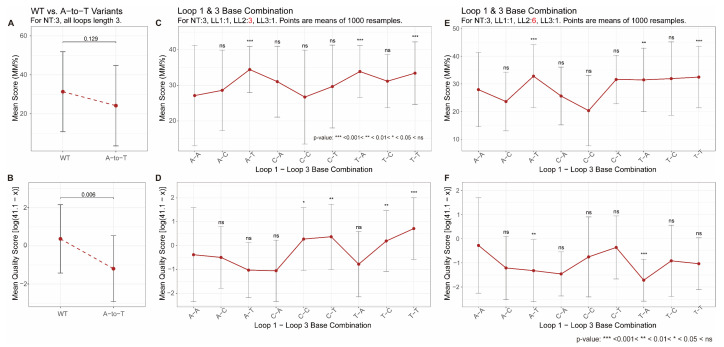
G4 stability score variation with loop composition. (**A**,**B**) Influence of adenine in human telomeric DNA repeats. Mean MM% from G4-Seq (**A**) and mean transformed quality score from G4-miner (**B**) are shown for wild-type (WT) telomeric G4 sequences ((GGGTTA)_n_) and sequences containing A-to-T variations. (**C**–**F**) Influence of non-guanine base composition in 1-nt first and third loops. Mean MM% (**C**,**E**) and transformed quality scores (**D**,**F**) are shown for three-layer G4s with 1-3-1 (**C**,**D**) and 1-6-1 (**E**,**F**) loop length configurations. All nine possible single-base combinations (A, C, or T in first loop × A, C, or T in third loop) were tested. The original G4-miner quality score (x) was transformed as ln(41.1 − x) in panels (**B**,**D**,**F**). Error bars represent standard deviations. Statistical significance was assessed using Wilcoxon rank-sum test: in (**A**,**B**), between WT and variant groups; in (**C**–**F**), between each combination and the reference A-A combination (indicated by symbols above data points). The terms LL1, LL2, LL3 represent lengths of loop 1, loop 2 and loop 3, respectively, and NT represents number of G-tetrads.

Regarding loop composition, we first tested the ability of these methods to detect the effect of A-to-T mutations in the evolutionarily conserved TTA loops of telomeric G4s (Figure 6A,B). Both G4-Seq and G4-miner correctly reflected the destabilizing influence of these mutations, as reported by Tucker et al. [73], with A-to-T mutant groups showing consistently lower stability scores than the wild-type. This successful detection is likely facilitated by the well-defined highly characteristic sequence and structure of telomeric G4s.

In contrast, both methods struggled to consistently recapitulate the base-specific effects observed in the 1-nt first and third loops by Guédin et al. [67]. When the central loop was 3-nt long (Figure 6C,D), G4-Seq detected the expected stability trend (A < C < T) only when the first loop was adenine, and correctly identified the destabilization by the A-A combination, but failed for other base combinations. G4-miner captured the A < C < T trend when the first loop was cytosine or thymine, but was insensitive when the first loop was adenine. With a 6-nt central loop (Figure 6E,F), G4-miner detected the destabilizing effect of adenine in specific comparative sets (e.g., C-A vs. C-C vs. C-T; T-A vs. T-C vs. T-T). Although both sequencing methods exhibit sensitivity to loop base composition, the resulting patterns are only partially consistent with biophysical observations. Given the well-documented, often conflicting results about loop composition effects in controlled biophysical experiments [43,64,67,70,71,72,73,74,75], it is plausible that the influence of loop composition is inherently variable and strongly context-dependent, and lacks consistent governing rules. Therefore, it is unsurprising that high-throughput sequencing methods, which integrate myriad contextual variables, face challenges in robustly detecting and reproducing these subtle, sequence-specific trends.

## 4. The Influence of Flanking Regions on G4 Structure and Stability

Within the genomic context, G4-forming sequences are invariably embedded within longer DNA strands, flanked by upstream and downstream nucleotides. These flanking regions, frequently omitted from in vitro studies, play a critical and multifaceted role in modulating G4 structure and stability.

### 4.1. Flanking Nucleotides as Modulators of G4 Folding

The addition of flanking nucleotides to a core G4 sequence can impart stabilizing effects. For instance, in studies of the *MYC* promoter G4 under physiological potassium concentrations (20 mM K^+^), isoforms retaining their natural flanking sequences exhibited T_m_ 2–3 °C higher than truncated core-only constructs [61]. This stabilization is often attributed to the flanking bases forming protective “capping” structures via stacking interactions with the terminal G-tetrads [78], or through specific interactions with the loop regions [79,80]. Subsequent research further suggested that such capping structures may facilitate the coordination of potassium cations, thereby enhancing stability [61].

Conversely, other studies report a destabilizing influence. A systematic investigation by Chen et al. on 371 distinct intramolecular G4s revealed that the addition of short 2-thymine flanking segments generally lowered T_m_ values. While the effect was typically modest, a dramatic decrease of 16.5 °C was observed in one case, challenging the universality of a stabilizing role for short flanking sequences [81]. Similarly, Bugaut and Alberti found that TTA flanking sequences on human telomeric G4s were destabilizing, with a more pronounced effect from the 5′ flank [82]. Zhang et al. also reported that 5′-flanking residues could prevent the formation of certain two-layer G4 conformations [83].

Beyond modulating stability, flanking sequences exert a profound influence on G4 topology. Chen et al. demonstrated that adding nucleotides, particularly at the 5′ end, strongly favors the formation of parallel topologies, often overriding the intrinsic propensity of a core sequence to form hybrid or antiparallel folds [81]. They proposed a mechanistic explanation: a 5′-end flanking nucleotide can disrupt a specific hydrogen bond with a 5′-terminal *syn* guanine, thereby destabilizing all folds that require this conformation and effectively selecting for parallel structures.

In addition to these general effects, specific flanking sequences can engender unique structural consequences. For example, Pavc et al. showed that a 5′ GC sequence can mediate G4 dimerization through the formation of an inter-quadruplex GCGC-tetrad, leading to stable higher-order assemblies [84]. Conversely, Do and Phan (2012) found that certain 5′ flanking residues could disrupt the stacking interactions necessary for dimerization, favoring monomeric G4 formation instead [85].

The recognition of this “flanking effect” necessitates a critical re-evaluation of the vast body of biophysical data acquired using truncated, minimal G4 constructs. While numerous NMR and crystal structures of such core sequences have revealed a rich polymorphism encompassing hybrid and antiparallel folds [86,87], The presence of a free 5′-terminus may itself be an experimental artifact that biases against parallel topologies. Consequently, the full spectrum of G4 structural diversity observed in vitro may not accurately reflect the in vivo landscape, where G4s are perpetually flanked by genomic sequences. From this perspective, high-throughput genomic methods, which analyze long DNA fragments with native flanks, might provide a more biologically representative view of predominant G4 topologies than some high-resolution structural studies of isolated cores.

### 4.2. Aligning the Evidence: The Flanking Effect in a Genomic Context

**Figure 7:**
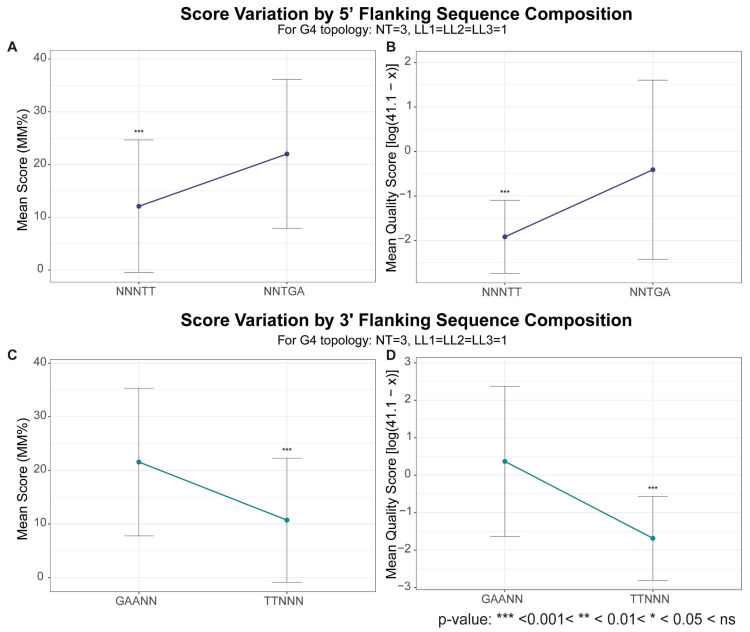
Influence of flanking sequences on G4 stability scores. (**A**,**B**) Effect of 5′-flanking sequences. Mean MM% from G4-Seq (**A**) and mean transformed quality score from G4-miner (**B**) are shown for three-layer G4s (all loops 1 nt) flanked at the 5′ end by either NNNTT or NNTGA. (**C**,**D**) Effect of 3′-flanking sequences. Mean MM% (**C**) and transformed quality score (**D**) are shown for three-layer G4s (all loops 1 nt) flanked at the 3′ end by either GAANN or TTNNN. The original G4-miner quality score (x) was transformed as ln(41.1 − x) in panels (**B**,**D**). Error bars represent standard deviations. Statistical significance between flanking sequence groups was assessed using the Wilcoxon rank-sum test. The terms LL1, LL2, LL3 represent lengths of loop 1, loop 2 and loop 3, respectively, and NT represents number of G-tetrads.

Given that flanking segments are an inherent feature of most genomic G4s, the influence of their nucleotide composition on G4 stability must be considered. Integrating evidence from Hatzakis et al. [61] and Chen et al. [81] reveals that specific flanking sequences can exert distinct effects on stability; for instance, 5′-TGA and 3′-GAA flanking sequences appear to stabilize G4s, whereas 5′- and 3′-TT sequences tend to be destabilizing. Although derived from different experimental systems, this distinction is consistently reflected in high-throughput data. Both the MM% values from G4-Seq and the transformed quality scores from G4-miner show clear differences between these groups: G4s flanked by TT sequences consistently exhibit significantly lower stability scores than those with stabilizing flanking sequences at both the 5′ and 3′ ends (Figure 7).

## 5. Conclusion and Outlook

### 5.1. Toward a Unified Understanding of Sequence-Stability Relationships

This review has systematically examined the intrinsic sequence features governing G-quadruplex (G4) thermostability and has endeavored to bridge observations from high-precision biophysical methods with those from high-throughput genomic assays. Overall, the two methodological paradigms yield consistent conclusions regarding the influence of most sequence features. For those trends showing incomplete agreement—such as the effects of loop permutation and base composition—the discrepancies primarily arise from their strongly context-dependent nature. In characterizing these particular features, both methodological paradigms exhibit instability in their conclusions, reflecting the inherent complexity and condition-specific variability of these structural determinants. The collective evidence allows for the synthesis of a set of generalized, albeit context-dependent, rules (Table 1).

The length and integrity of the G-tracts establish the foundational stability of the G-quadruplex. Generally, the correlation between G-tract length and G4 stability is consistently observed across methodological paradigms, though it does not follow a simple monotonic function. Furthermore, the destabilizing effect of bulges—including the influence of bulge size—is also captured by both experimental approaches. However, the influence is modulated by additional complex factors, such as the conformational strain imposed by short loops, which underlies the “anomalous stability” of certain configurations, and the capacity of long bulges to form stabilizing secondary structures.

The well-established inverse correlation between loop length and G4 stability is consistently observed across both biophysical and high-throughput methodological paradigms. In contrast, the effects of loop permutation show only partial consistency between these approaches, likely due to the intrinsic context-dependency of this structural feature combined with the attenuated sensitivity of sequencing-based methods to loop rearrangement. Furthermore, the influence of loop nucleotide composition demonstrates marked context-dependence. While both methodological frameworks detect the stabilizing role of adenine in canonical telomeric G4 structures, they fail to establish clear, generalizable rules regarding base-specific effects in more diverse sequence contexts.

Evidence suggests that the base composition of flanking sequences can modulate stability, with certain motifs showing consistent effects across experimental approaches. Critically, as an inevitable component of genomic G4s, flanking sequences appear to exert a structural influence that favors parallel topologies, which should not be ignored especially when discussing G4 conformation and stability under genomic context.

### 5.2. Toward Quantitative and Predictive Models

The rules summarized in this review remain largely qualitative or semi-quantitative. For instance, we can reasonably predict that a sequence featuring short loops, continuous G3-tracts, no bulges, and specific 5′ flanking bases will form a highly stable parallel G4, yielding a strong signal in G4-Seq assays. Conversely, a sequence with long loops, a central bulge, and short G2-tracts is likely to adopt an unstable, transient structure associated with a weak G4-Seq signal.

However, translating these qualitative relationships into a fully quantitative and predictive framework represents a critical objective for future research. Achieving this will require innovative experimental strategies, such as the construction of a synthetic oligonucleotide library comprising thousands of rationally designed G4 sequences that systematically vary key structural features. Based on parallel analyses using both high-throughput biophysical profiling and next-generation sequencing-based methods, such a design would enable the direct mapping and calibration of sequencing-derived metrics—such as mismatch rates or quality scores—against precisely measured thermodynamic parameters. Such effort would pave the way for establishing a foundational “conversion table” or computational model capable of bridging observations across methodological scales.

### 5.3. Unresolved Questions and Concluding Remarks

Despite considerable progress in deciphering the principles of G4 stability, several fundamental questions remain open. What is the precise quantitative relationship between the kinetics of polymerase pausing—as detected in high-throughput assays—and the thermodynamic stability of the underlying G4 structure? To what extent do the numerous G4-binding proteins in the cellular milieu modulate or override these intrinsic sequence-stability rules? Furthermore, do RNA G4s and DNA–RNA hybrid G4s follow the same structural principles as their DNA counterparts?

From a methodological perspective, significant limitations persist. While high-throughput approaches can statistically capture the influence of major structural features on stability, their analytical power is constrained. For instance, when controlling for variables—such as examining perfect three-layer G4s with 1-nt loops—many specific sequence combinations are either absent from the human genome or occur too infrequently to support robust statistical analysis. Consequently, this review has necessarily focused on more prevalent sequence features. Similarly, the biophysical studies we have integrated are predominantly those that systematically and batch-test the effects of specific sequence variations.

In conclusion, the thermostability of a G-quadruplex is not dictated by any single sequence element but emerges from the complex interplay between its G-tract core, loop architecture, and flanking environment. By systematically deconstructing these features and understanding their roles in analytical methods of different precision and throughput, we are beginning to align experimental evidence from complementary domains. This synthesis of knowledge is a crucial prerequisite for unlocking the ability to predict—on a genome-wide scale—where functionally significant G4s reside, how stable they are, and what roles they fulfill in cellular physiology and disease.

## Figures and Tables

**Table 1:** Alignment of evidence from high-precision biophysical experiments and high-throughput sequencing data.

Sequence Feature	Patterns from Biophysical Studies	Consistency with High-Throughput Data	Key Observations from High-Throughput Data
G-Tract Length	Stability generally increases with length, though non-monotonically; anomalous stability observed for 3-layer G4s with 1-nt loops [40]	Generally consistent (Both methods, Figure 2).	1. G4-miner capturing all trends accurately 2. G4-Seq showing discrepancies for 5-layer G4s with longer loops
Bulges	Bulges are generally destabilizing; the effect is size-dependent [45,48]; compensatory stabilization was reported [49].	Consistent (Both methods, Figure 3)	1. Both methods detecting destabilization by bulges 2. Negative correlation for bulges < 5 nt suggesting destabilizing effect of bulge size 3. Positive correlation for bulges > 5 nt suggesting compensatory structural mechanisms
Total Loop Length	Strong inverse correlation with stability was observed [53].	Consistent (Both methods, Figure 4)	1. Robustly captured by both G4-Seq and G4-miner
Loop Permutation	Significant impact on stability and topology was observed; central loop length plays dominant role [62,64]	Partially Consistent (Figure 5)	1. Partial agreement for unequal loop lengths x≠y≠z 2. G4-miner better capturing loop combina-tions x=y≠z 3. Reflecting context-dependency of bio-physical observations and attenuated sensitivity of sequencing-based methods to loop order
Loop Base Composition	The effect is highly context-dependent; adenine can be stabilizing or destabilizing depending on structural context [67,70,71,72,73]	Partially Consistent (Figure 6)	1. Both methods exhibiting sensitivity to loop composition 2. Both methods detecting stabilizing effect of adenine in telomeric G4s 3. Limited consistency for G4s in more diverse sequence contexts
Flanking Regions	The effect is composition-sensitive; specific flanking sequences differentially modulate G4 stability, with 5′-TGA/3′-GAA conferring stabilization [61] and 5′-TT/3′-TT producing destabilization [81].	Consistent (Both methods, Figure 7)	1. Both methods confirming differential effects of specific flanking motifs

## Data Availability

The data presented in this study are available in Gene Expression Omnibus at http://www.ncbi.nlm.nih.gov/geo/query/acc.cgi?acc=GSE63874 (accessed on 1 July 2025) and https://www.ncbi.nlm.nih.gov/geo/query/acc.cgi?acc=GSE159307 (accessed on 1 July 2025), reference number 8 and 26 respectively.

## Appendix A. Data Sources and Processing

The G4-Seq data for the Na^+^ vs. K^+^ conditions were obtained from Chambers et al. [8], and the G4-miner data were sourced from Tu et al. [30]. Both datasets were processed using the respective workflows described in the original publications. Potential G4 (pG4) loci were identified using pqsfinder [9], and downloaded from https://pqsfinder.fi.muni.cz/hub/hg19/pqsfinder_hg19_gff.tar.gz (accessed on 10 September 2025). For each pG4 locus, stability levels were assigned based on the maximum Mismatch Percentage (MM%) values from G4-Seq or averaged sequencing quality scores from G4-miner, calculated within a 50 bp window centered on the locus and restricted to the same strand.
